# Establishment and characterization of a novel patient-derived *TP63*-rearranged anaplastic large-cell lymphoma model PTCL-S1

**DOI:** 10.1007/s13577-025-01264-1

**Published:** 2025-07-26

**Authors:** Kelila Xin Ye Chai, Elizabeth Chun Yong Lee, Zhimei Li, Nur Ayuni Binte Muhammad, Jing Quan Lim, Dachuan Huang, Soon Thye Lim, Jason Yongsheng Chan, Choon Kiat Ong

**Affiliations:** 1 Lymphoma Translational Research Laboratory, Division of Cellular and Molecular Research, National Cancer Centre Singapore, 30 Hospital Blvd, Singapore, 168583 Singapore; 2 Cancer Discovery Hub, National Cancer Centre Singapore, Singapore, Singapore; 3 Division of Medical Oncology, National Cancer Centre Singapore, 30 Hospital Blvd, Singapore, 168583 Singapore; 4 Duke-NUS Medical School, Singapore, Singapore

**Keywords:** Anaplastic large cell lymphoma (ALCL), TP63 rearrangement, TP63-TBL1XR1 fusion, Tumour models, Patient-derived xenograft

## Abstract

Anaplastic large-cell lymphoma (ALCL) accounts for 15% of all peripheral T-cell lymphomas globally and can be further divided into subcategories, of which patients with ALK-negative ALCL have dismal prognosis and overall survival. We established a patient-derived xenograft (PDX) and in vitro model (designated PTCL-S1) of *TP63*-rearranged ALK-negative ALCL from the primary tumour site of a 55-year old Chinese woman. Whole genome sequencing of the patient’s tumour identified various mutations including *AKT1* and *NOTCH1*, as well as the *TP63*-*TBL1XR1* gene fusion. RNA sequencing followed by Sanger sequencing confirmed the gene rearrangement in original tumour, PDX and PTCL-S1 cell line. Immunohistochemistry profiling of the PDX model and cell-line were consistent with the patient’s primary tumour sample (CD3 + /CD30 + /CD79a-). Cytotoxic agents (doxorubicin, etoposide and gemcitabine) commonly used in ALCL treatment exhibited potent anti-proliferative activity in the cell-line. In conclusion, the established PTCL-S1 cell line can be a useful tool for further investigation of the understanding of *TP63*-rearranged ALK-negative ALCL.

**Supplementary Information:**

The online version contains supplementary material available at 10.1007/s13577-025-01264-1.

## Introduction

Systemic anaplastic large-cell lymphoma (ALCL) belongs to a family of nodal peripheral T-cell lymphomas (PTCL) that are rare globally [1]. Conventionally, ALCL is divided into ALK (anaplastic lymphoma kinase)-positive and ALK-negative subtypes with divergent pathobiology and clinical phenotype [2]. While ALK-positive ALCL is generally defined by good response to conventional chemotherapy and favourable prognosis, ALK-negative ALCL belongs to a heterogeneous subgroup that is poorly characterized, with widely disparate clinical outcomes [3, 4]. Notably, ALK-negative ALCL may be further stratified into distinct molecular subtypes based on genomic rearrangements of *DUSP22* and *TP63*. These molecular alterations are mutually exclusive and make up a minority of ALK-negative ALCL, accounting for between 13 and 30% and 2–8% cases, respectively [3, 5–7]. While *DUSP22* rearrangement has been associated with survival outcomes of up to 90% at five years, *TP63* rearrangement has been shown to portend a dismal prognosis [3, 5].

Contemporary anthracycline-based “CHOP” or “CHOP-like” regimens are typically used in the first-line treatment of ALCL, regardless of known molecular alterations. The incorporation of brentuximab vedotin, a CD30-targeted antibody–drug conjugate, is also approved in this setting, following the pivotal ECHELON-2 study [8], though outcomes remain poor for ALK-negative/*TP63*-rearranged cases. The urgent need for better understanding of the disease pathobiology and for improved therapeutic strategies in *TP63*-rearranged ALCL is however, hampered by the lack of ex vivo model systems for mechanistic studies and evaluation of drug candidates.

In this study, we established a novel patient-derived murine xenograft and cell-line model of ALK-negative ALCL with a *TP63* rearrangement (designated PTCL-S1). We characterized the genomic profile of this model via whole genome sequencing and RNA sequencing, and examined ex vivo responses to a panel of conventional chemotherapeutic agents.

## Materials and methods

### Clinical biospecimen and data acquisition

Clinical information was obtained from hospital electronic medical records. Basic demographic data including sex, age and ethnicity of the index patient was checked against her National Registry Identification Card. All histological parameters were reviewed by expert haemato-pathologists. Written informed consent from the patient for use of clinical data and biospecimens was obtained in accordance with the Declaration of Helsinki. Ethics approval from the SingHealth Centralized Institution Review Board (CIRB 2018/3084) was obtained for tissue collection and patient consent protocols.

### Clinical and pathological description of the patient

**Fig. 1:**
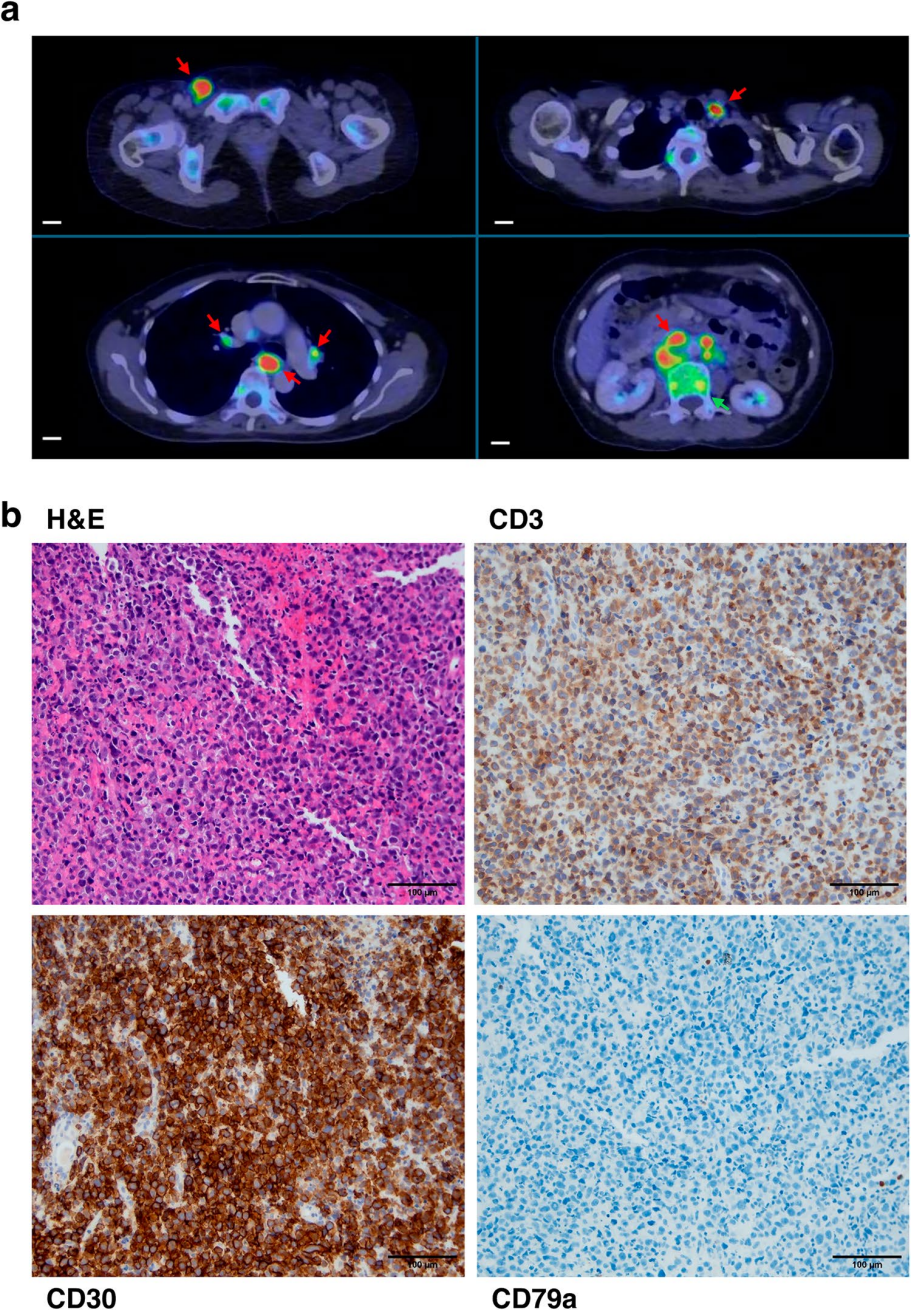
Clinical and pathological features of patient with ALK-negative ALCL. **a** 18F-fluorodeoxyglucose Positron Emission Tomography/Computed Tomography (FDG-PET/CT) imaging taken at time of relapse showed hypermetabolic supradiaphragmatic and infradiaphragmatic lymphadenopathy (red arrows), including the right inguinal, left supraclavicular, subcarinal, peri-hilar and para-aortic regions, as well as FDG-avid bony lesions indicative of marrow infiltration (green arrow) (scale bar: 2 cm). Transverse diameter of dominant nodes: (Top Left to Right) 2–1.2 cm (Bottom Left to Right) 1.7–5 cm. **b** IHC of patient tumour tissue revealed to be strongly and uniformly positive for CD30 and CD3, while being negative for CD79a (scale bar: 100 µm).

A 55 year old Chinese woman was diagnosed with stage 2 anaplastic lymphoma kinase (ALK)-negative anaplastic large cell lymphoma (ALCL) of the posterior nasopharynx. She had presented with intermittent nasal discharge and epistaxis over a 3 month duration, associated with right-sided hearing impairment, blocked nose and unintentional weight loss of 5 kg. 18F-fluorodeoxyglucose Positron Emission Tomography/Computed Tomography (FDG-PET/CT) imaging showed a FDG-avid mass in the posterior nasopharynx extending into the skull base and sphenoid sinus, as well as FDG-avid cervical lymph nodes bilaterally. Biopsy of the posterior nasopharyngeal mass showed negativity for ALK staining on immunohistochemistry. In addition, fluorescence in situ hybridization for *ALK* and *DUSP22* rearrangements were both negative. Large tumour cells with nucleolated, rounded and irregular hyperchromatic nuclei, enclosed in ample, pale cytoplasm were observed, staining strongly positive for CD30, CD3 and CD2. EBV-encoded small RNA (EBER), ALK, CD20, CD79A, CD10, CD56, CD123 and TCL-1 were all negative. Ki-67 was positive in 70–80% of tumour cells. She achieved complete remission following six cycles of induction ICE (ifosfamide, carboplatin, etoposide) chemotherapy without consolidation with autologous stem cell transplantation (ASCT). However, the patient eventually relapsed 5-years later, presenting with involvement of lymph nodes above and below diaphragm, bone marrow, and cerebrospinal fluid. Excision of the right inguinal node and bone marrow biopsy confirmed relapse of ALK-negative ALCL (Fig. 1). She was treated with four cycles of CHOP (cyclophosphamide, doxorubicin, vincristine, prednisolone) and two cycles of high-dose methotrexate, achieving complete metabolic remission, followed by consolidation ASCT. Unfortunately, she relapsed approximately one month post-engraftment and passed away shortly.

### Establishment of patient-derived xenograft (PDX)

Primary tumour tissue before chemotherapy from the patient was dissected and implanted into the right flank of NSG mice subcutaneously. Measurements were taken twice a week and passaged when tumour volume hit > 1000 cm^3^, but not exceeding 2000 cm^3^. The PDX was considered established after it had been successfully passaged for 4 generations. Xenograft studies were conducted in compliance with animal protocols approved by the SingHealth Institutional Animal Care and Use Committee (IACUC).

### Establishment of patient-derived cell line

Tissue from the established PDX was digested and homogenized using human tissue dissociation kit (Miltenyi Biotec, Bergisch Gladbach, Germany) following the manufacturer’s protocol. PTCL-S1 was maintained in Dulbecco's Modified Eagle Medium (DMEM) supplemented with 10% foetal bovine serum (FBS), 10% horse serum (HS), 2 mM L-glutamine and 100 units/ml of Penicillin/Streptomycin (Thermo Fisher Scientific, Waltham, MA, US) in a humidified incubator at 37 ℃, 5% CO_2_. Culture medium was changed every 3–4 days and mouse cell depletion was done twice, at passage 5 and passage 10, using Mouse Cell Depletion Kit (Miltenyi Biotec, Singapore). Cells were maintained as described for more than 60 passages.

### Short tandem repeat (STR) analysis

PTCL-S1 cells were collected and sent for standard STR genotyping analysis (Axil Scientific, Singapore) to establish a unique genomic fingerprint of the established cell line as well as to check for any cross-contamination.

### Cell proliferation assay

Cell proliferation was measured by cell counting with the automated cell counter Countess II (Thermo Fisher Scientific, Waltham, MA, US). PTCL-S1 cells were seeded at 3 × 10^5^ cells/well in 24-well plate in triplicate and cultured for 0, 24, 48 and 72 h. Cells were counted at each time point and doubling time was calculated using the formula of $\frac{Tln(2)}{\text{ln}(\frac{Xe}{Xb})}$ where T = time in hours, e = final cell number and b = initial cell number. Characterization of drug response in PTCL-S1 was performed using cytotoxic drugs that are commonly used in clinical practice as part of standard treatment regimens, including etoposide, gemcitabine and doxorubicin (Selleck Chemicals, USA). Drug cytotoxicity was tested by seeding 2 × 10^4^ PTCL-S1 cells per ml media in 24-well plates and treated with various concentrations of each drug. Cells were incubated for 72 h and then resuspended and 100 µl of cells were plated in 96-well plate in triplicates. Cell viability was assessed using Promega CellTiter-glo following manufacturer’s instructions (Promega, Madison, WI, US) and measuring the absorbance at 480 nm using Tecan M200 Infinite 96-well plate reader (Tecan, Männedorf, Switzerland). Cell viability was calculated as a percentage of control absorbance and IC50 values were estimated using GraphPad Prism 9 (GraphPad Software, CA, USA).

### Immunohistochemistry

Histological investigation was performed on 4 µm thick sections of a representative formalin-fixed paraffin-embedded (FFPE) tumour and cell line samples. Tumour from the PDX was fixed in 10% neutral-buffered formalin and made into FFPE blocks for validation using immunohistochemistry. The sections were deparaffinized and stained with haematoxylin and eosin (H&E) as well as antibodies against CD3, CD30 and CD79a following standard protocols in the clinical laboratory of the Singapore General Hospital. Immunohistochemistry profiles of the xenograft and cell line were compared with the original tumour.

### RNA extraction, RNA sequencing and RT-PCR for fusion detection

Cells were collected and RNA were isolated from PTCL-S1 using Qiagen RNeasy Mini Kit (Qiagen, Valencia, CA, USA). RNA was quantified using NanoDrop 2000 Spectrophotometer (Thermo Fisher Scientific, MA, USA) and the integrity of RNA was determined by electrophoresis using the 2100 Bioanalyzer (Agilent Technologies, USA). Reverse transcription polymerase chain reaction (RT-PCR) was performed using BioRad iScript cDNA synthesis Kit according to manufacturer’s instructions (Bio-Rad Laboratories, Hercules, CA, USA). Whole transcriptome sequencing was performed on the MGI G400 platform (MGI Tech, China) using standard manufacturer’s protocol. The reads were aligned to the human genome hg19 RefSeq reference transcriptome by STAR [9] and fusion detection was performed using CICERO v1.9.6 [10].

### DNA extraction and whole genome sequencing

Genomic DNA was extracted using Qiagen DNeasy Blood & Tissue kit (Qiagen, Valencia, CA, USA) following manufacturer’s instructions and quantified using NanoDrop 2000 Spectrophotometer (Thermo Fisher Scientific, MA, USA). Whole genome sequencing was performed on the Illumina HiSeq X Ten platform as paired-end 150-base pair reads (Macrogen, Singapore).

### Mutational variant-calling on DNA sequencing reads

DNA sequencing adaptors, trailing low-quality bases and polyG sequences were removed by fastp (v0.19.6) [11]. The pre-processed reads were aligned using BWA-MEM (v0.7.17) [12] to the hs37d5 human reference genome. Polymerase chain reaction (PCR) duplicates were subsequently marked by Sambamba (v0.6.5) [13]. The alignment coverage statistics were computed by Qualimap [14] (v2.2.1). Short-variants were called using Strelka2 (v2.9.4). wAnnovar (November 2020) [15] was used to annotate the short-variants. The filtering criteria on the short-variants were further filtered against low complexity regions, dbSNP(v132) positions without COSMIC IDs, simple repeats and homopolymers as described in JQ Lim et al., 2022 [16]. Structural rearrangements (SR) were called using Manta (v1.6.0) [17] and annotated by annotSV (v1.2). [18]. Each candidate SR was subjected to the following filtering criteria: (1) SR is supported by at least 3 discordant read-pairs and at least 3 soft-clipped reads, and the sum of all supporting reads is at least 10; (2) at least 10 × coverage in both tumour and matching-normal data; and (3) SR is at least 1000 bp in size.

### Validation of somatic mutations and gene fusion by Sanger sequencing

Specific flanking primer pairs for the selected mutations and fusion product to be validated were designed using Primer-BLAST and the primer sequences are as per specified.

NOTCH1-Fwd: 5’-GAGTAGCTGTGCTGCGAGG-3’.

NOTCH1-Rev: 5’-AACCAATACAACCCTCTGCG-3’.

AKT1-Fwd: 5’-GAGGCCAAGGGGATACTTACG-3’.

AKT1-Rev: 5’-TTCTGTCGCTGGCCCTAAGA-3’.

TP63-TBL1XR1-Fwd: 5’-CCCCAGCTCATTTCTCTTGGAA-3’.

TP63-TBL1XR1-Rev: 5’-GGCAAACCCATCATAGGAACC-3'.

TBL1XR1-TP63-Fwd: 5’-CTGTGCCTGGAACCCTGTT-3’.

TBL1XR1-TP63-Rev: 5’-CGCGTGGTCTGTGTTATAGG-3’.

Two primer pairs were used to detect the TP63-TBL1XR1 gene fusion products. PCR was performed by using 1 st Base REDiant II PCR master mix (Axil Scientific, Singapore) following manufacturer’s instructions. The resulting amplicons were sent out for Sanger sequencing by 1 st Base (Axil Scientific, Singapore) and analysed.

### Data availability

All data, xenograft and cell lines that support the findings of this study are available from the corresponding authors upon reasonable request.

## Results

### Establishment and characterization of patient-derived xenograft (PDX) model

**Fig. 2:**
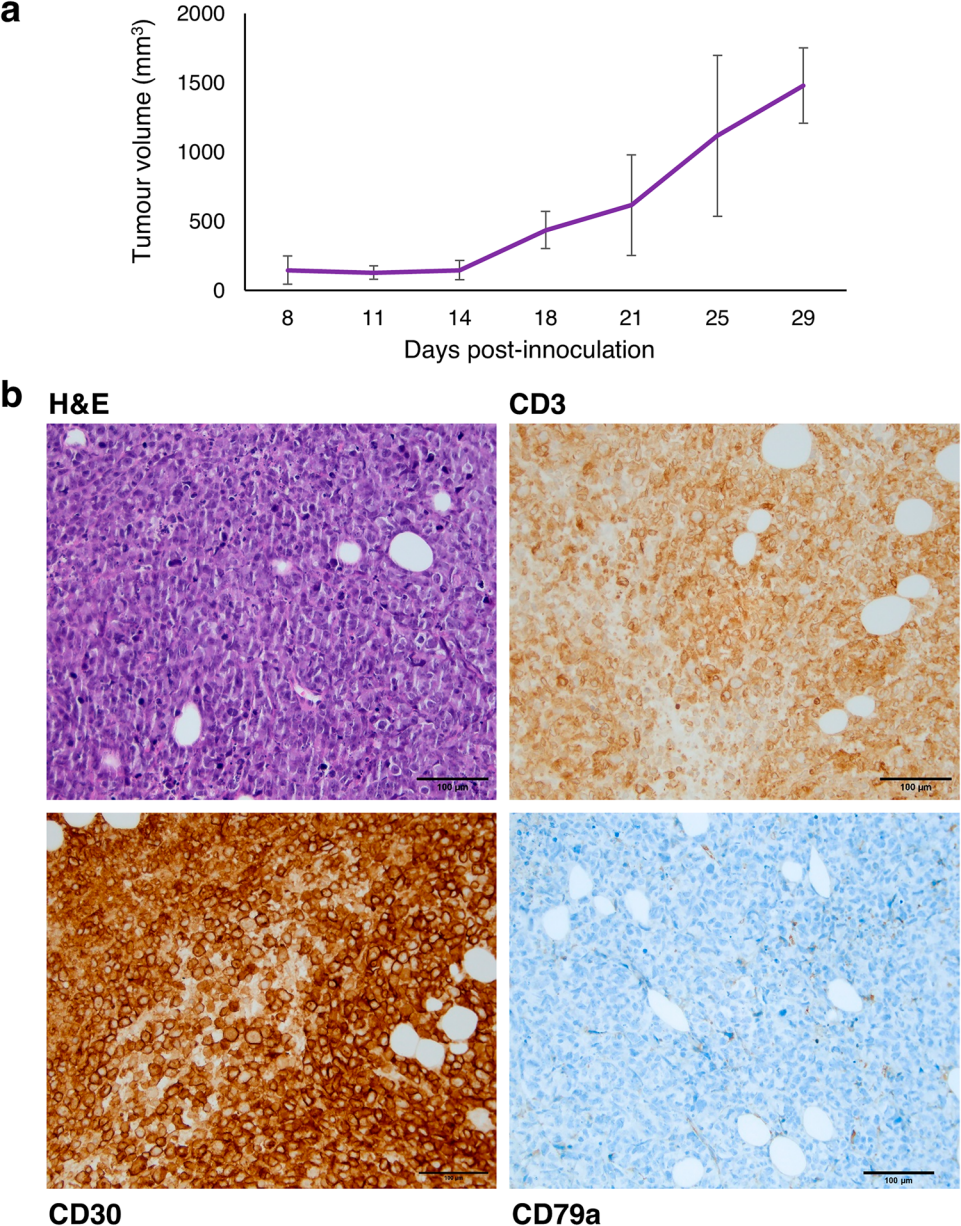
PDX establishment from patient tissue. **a** Solid lymphoma developed 8 days following subcutaneous implantation of tumour tissue into NSG mice. **b** IHC of established PDX revealed similar characteristics to the original patient tumour (scale bar: 100 µm)

Patient tumour was dissected and subcutaneously injected into NSG mice, with solid lymphoma tissue developing after 8 days of transplantation (Fig. 2a). IHC of the harvested tumour after 4 generations revealed similar characteristics to the original patient tumour, i.e. positive for CD3, CD30 and negative for CD79a (Fig. 2b) This indicates that the xenograft can propagate and retain the characteristics of the original tumour.

### Establishment and morphological characterization of patient-derived cell-line PTCL-S1

**Fig. 3:**
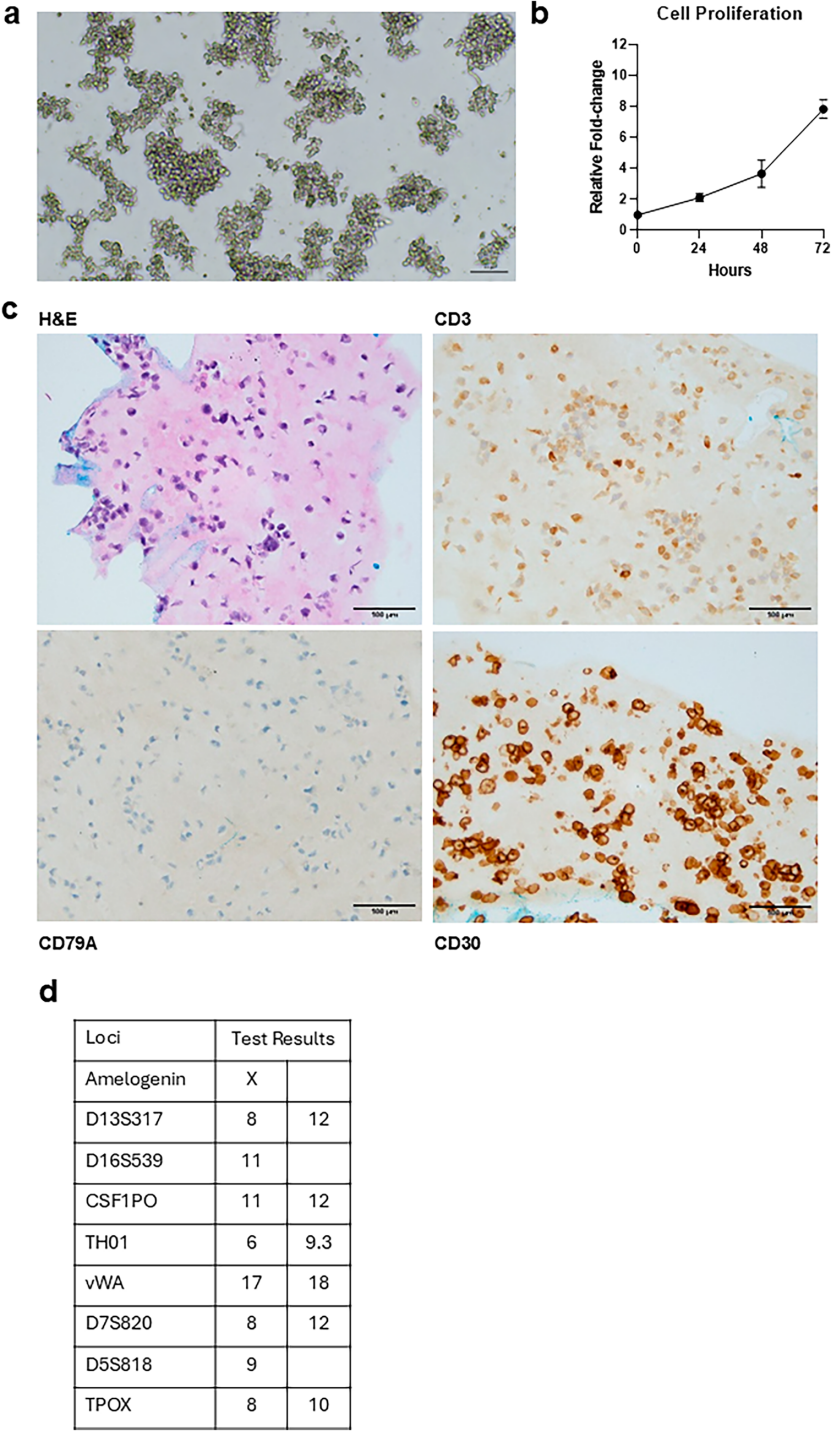
Characterization of PTCL-S1 cell-line. **a** Brightfield image of PTCL-S1 at 10 × magnification (scale bar: 100 µm) at passage 31. PTCL-S1 cells are mostly spherical with some irregularities in their shape, appearing to be elongated, and they tend to grow in clumps. **b** PTCL-S1 cells grow readily in RPMI-1640 supplemented with 10% FBS, 10% HS and has an estimated doubling time of 22.26 ± 0.82 h. **c** IHC of established cell line at passage 10. IHC revealed that PTCL-S1 highly expresses CD30 and CD3 and is negative for CD79a (scale bar: 100 µm). **d** STR profiling of PTCL-S1 demonstrated a unique DNA fingerprint of the cell line, with no evidence of cross-contamination with cell-lines from known databases

The cell line PTCL-S1 was derived from the established PDX tissue and the cells grew in suspension for more than 60 passages. PTCL-S1 are mostly spherical, with some irregularities in shape where some cells appear to be elongated, and they tend to grow in clumps (Fig. 3a). They have a doubling time of 22.26 ± 0.82 h (Fig. 3b) and are interleukin-2 (IL-2) independent. Further IHC investigations were conducted and compared with both original patient tissue and PDX tissue. The results revealed that PTCL-S1 highly expresses CD30 and CD3, and is negative for CD79a (Fig. 3c), which is concordant with the patient tumour and PDX tissue. STR profiling demonstrated PTCL-S1’s unique DNA fingerprint, with no matches to any cell-line from known databases (Fig. 3d).

### Genomic characterization of PTCL-S1

**Fig. 4:**
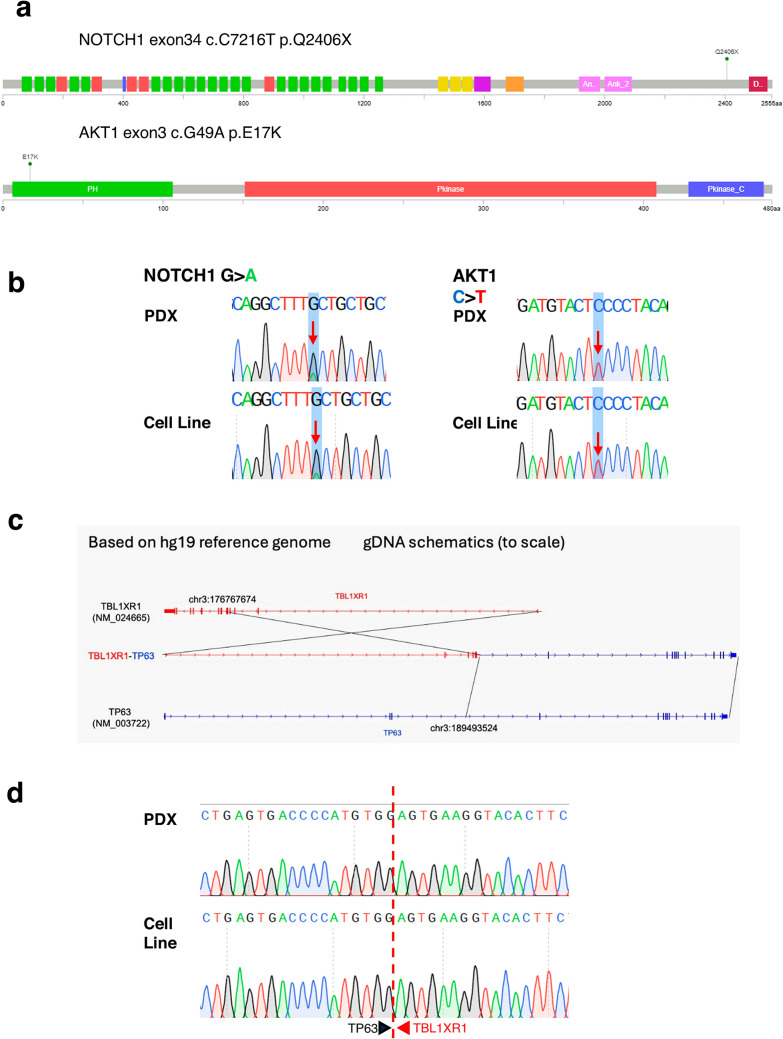
Genomic characterization of PTCL-S1. Validation of genomic characteristics in PDX and cell line was performed by sanger sequencing at passage 4 and 58 respectively. **a** *NOTCH1* and *AKT1* somatic mutations were detected by Whole Genome Sequencing (WGS). **b** Both somatic mutations were verified and validated in the PTCL-S1 PDX and cell-line by Sanger sequencing. **c** TP63-TBL1XR1 fusion was identified following WGS of the primary tumour and RNA-Seq of PTCL-S1 cell-line passage 40. **d** Verification and validation of the TP63-TBL1XR1 fusion in the PTCL-S1 PDX and cell-line by sanger sequencing

The patient-matched bulk tumoural tissue and non-tumoural blood are whole-genome sequenced and generated a total of 224.7 Gbp (Q30, read 1: 91.5%, read 2: 68.2%) and 110.7 Gbp (Q30, read 1: 85.2%, read 2: 70.9%) of data, respectively. After alignment to the human reference genome, the libraries achieved an effective PCR duplicated coverage of 61.5 × and 31.2x, respectively (Supplementary Table 1). Genomic investigation of patient tumour using Whole Genome Sequencing (WGS) detected a series of heterozygous mutations with variant allele frequencies (VAF) between 0.1 and 0.27, of which 5 nonsynonymous mutations *FAT3* (c.C12623T; VAF 0.27)*, NRG1* (c.C929T; VAF 0.21), *SLFN5* (c.C1064T; VAF 0.21), *CLRN1* (c.T275A; VAF 0.17), *AKT1* (c.G49A; VAF 0.13), 1 frameshift deletion *TNRC6C* (c.1837delA; VAF 0.26) and 1 stop-gain mutation *NOTCH1* (c.C7216T; VAF 0.10) were observed. We then validated the mutations in 2 genes, namely *NOTCH1* (c.C7216T) and *AKT1* (c.G49A) (Fig. 4a), in the PDX and PTCL-S1 cell line (Fig. 4b) using Sanger sequencing. These mutations were concordant across all samples. WGS of the patient tumour also detected the fusion of *TP63*-*TBL1XR1*, showing a balanced inversion of *TP63* and *TBL1XR1* (Fig. 4c.) Sanger sequencing of the cDNA from PDX and the cell line verified that the fusion product was present (Fig. 4d).

### In vitro* characterization of responses to conventional cytotoxic agents*

**Fig. 5:**
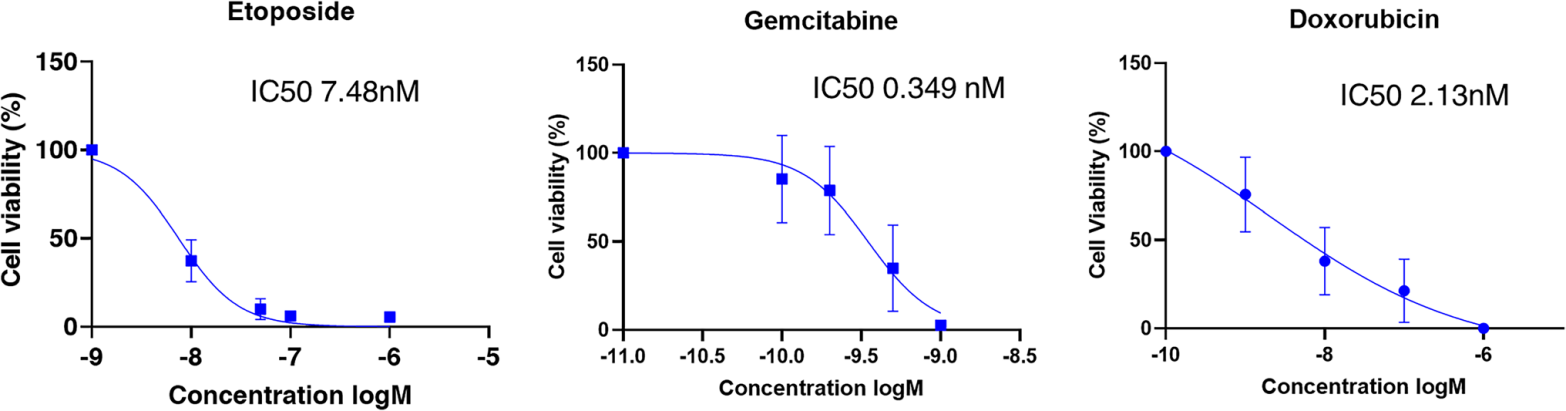
Drug response characterization of PTCL-S1 to standard PTCL treatment regimens. PTCL-S1 treated with the various drugs showed reduction of viability in a dose-dependent manner with reasonable sensitivity to each drug. All drug response experiments were performed using cells between passage 30–40

Following morphological and genomic characterization of PTCL-S1, we investigated the drug response of PTCL-S1 to conventional PTCL treatment regimen (Fig. 5), such as Etoposide, Gemcitabine and Doxorubicin. The cells were incubated with different concentrations of the respective drugs for 72 h before performing ATP assay to assess their drug sensitivity and establishing the IC50. PTCL-S1 demonstrated a reduction in cell viability following drug treatment in a dose-dependent manner and showed potent sensitivity to all selected drugs with an IC50 of 7.48 nM, 0.349 nM and 2.12 nM to etoposide, gemcitabine and doxorubicin respectively.

## Discussion

ALCL is a subset of PTCL derived from Th17 cells with the characteristic expression of IL-17A and IL-17F, and is uniformly CD30-positive in nature [19, 20]. ALCL can be further characterized into ALK-negative and ALK-positive which can affect disease prognosis. ALK-negative patients typically perform worse [20] and are more prone to relapses when compared with ALK-positive patients, at 49% and 43% for 5 year overall survival (OS) and progression-free survival (PFS) respectively [21]. There is a need to understand the molecular and genetic features of ALK-negative ALCL to help improve patient outcome and understanding of the disease.

Chromosomal rearrangements seen exclusively in ALK-negative ALCL patients include *TP63* rearrangement, which results from a *TP63*-*TBL1XR1* intrachromosomal inversion [5, 22] and *DUSP22* rearrangement. Patients harbouring *TP63* rearrangements perform worse overall where *DUSP22*-rearranged patients in the study by Pedersen et al. had an 80% 5 year OS but the two patients with *TP63* rearrangements both died within 2 years of diagnosis [5]. Another report by Parrilla Castellar et al. reported 90% 5 year OS for *DUSP22*-rearranged patients when compared with only 17% OS in *TP63*-rearranged patients [3]. Meanwhile, the sole case of *TP63*-rearranged patient in the study by Hapgood died within 6 months of diagnosis [23].

ALK-negative ALCLs are difficult to study due to their genetic and biological heterogeneity, and there are unique subtypes within this classification. Cases with abnormal immunophenotypes can result in confusion with other neoplasms [24]. In one case, fluorescence in situ hybridization (FISH) was unable to identify the *TP63*-rearrangement and chromosomal rearrangements were only picked up by RNA-seq [25].

According to our knowledge, other than the 21 ALCL cell models described in the 2004 report by Drexler and MacLeod [26], there are a total of 29 published ALCL cell models now. However, many are lost or do not have sufficient information on the genetic and molecular features as shown in Table 1. Of which, only 10 are known to be ALK-negative, with only 7 available from a commercial cell bank. There is currently only 1 cell line, DL-40, which was first reported in 1990 [27], and then subsequently found to harbour the TP63-TBL1XR1 gene fusion using RNA-seq [28]. DL-40 was originally derived from the peripheral blood of a case in keeping with aggressive ALCL in a terminal leukemic phase, whereas our current PTCL-S1 model is derived from a primary tumour at an earlier disease state upon diagnosis and before chemotherapy. The clinical implications of *TP63*-rearrangements have been widely discussed in previous studies as mentioned and the addition and establishment of new in vitro models, particularly those that harbour gene rearrangements, are important to further our study of the disease.

**Table 1:** ALCL cell lines in publications

Cell line	Published Year	Patient age and sex	Sample Site	ALK status	Translocation	Cellosaurus accession number	Availability	References
DL-40	1990	64F	Peripheral Blood	–	TP63-TBL1XR1	CVCL_2889	JCRB	[27, 28]
TK-ALCL1	2024	59 M	Lymph Node	+	NPM-ALK	CVCL_E2T4	Authors	[31]
AMS3	1994	23 M	Tumour	+	NPM-ALK	CVCL_H629	?	[32]
CHIC	2011	32 M	Cerebrospinal fluid	+	NPM-ALK	CVCL_E2T5	Authors	[33]
COST	2004	4 M	Peripheral Blood	+	NPM-ALK	CVCL_9491	?	[34]
DEL	1990	12 M	Pleural Effusion	+	NPM-ALK	CVCL_1170	DSMZ	[35]
HSC-M1	2001	5F	Bone Marrow	+	NPM-ALK	CVCL_H630	Lost	[36]
JB6	1990	12 M	Peripheral Blood	+	NPM-ALK	CVCL_H633	?	[37]
Karpas-299	1988	25 M	Peripheral Blood	+	NPM-ALK	CVCL_1324	DSMZ	[38]
Ki-JK	1993	15 M	Pleural Effusion	+	NPM-ALK	CVCL_2093	DSMZ/JCRB	[39]
L-82	2002	24F	Pleural Effusion	+	NPM-ALK	CVCL_2098	DSMZ	[40]
SU-DHL-1	1974	10 M	Pleural Effusion	+	NPM-ALK	CVCL_0538	ATCC/DSMZ	[41]
SR/SR786	1988	11 M	Pleural Effusion	+	NPM-ALK	CVCL_1711	ATCC/DSMZ/NCI-DTP	[42]
SUP-M2	1989	5F	Cerebrospinal fluid	+	NPM-ALK	CVCL_2209	DSMZ	[43]
UCONN-L2	1995	?	Lymph Node	+	NPM-ALK	CVCL_A693	?	[44]
FE-PD	1994	46F	Peripheral Blood	–	MKLN1-AS1-DUSP22	CVCL_H614	Authors	[28, 45]
*MAC-2A*	1989	47 M	?	–	PCM1-JAK2	CVCL_H637	DSMZ	[28], [46]
TLBR-1	2010	42F	Tumour	–	–	CVCL_L177	DSMZ	[47]
TLBR-2	2012	43F	Tumour	–	–	CVCL_A1EY	DSMZ	[48]
TLBR-3	2012	45F	Tumour	–	–	CVCL_A1EZ	DSMZ	[48]
TLBR-4	2017	?	Tumour	–	–	CVCL_A1FA	DSMZ	[49]
DL-95	1998	40 M	?	?	?	CVCL_IR15	?	[50]
JK	1999	58 M	Tumour	–	?	CVCL_H635	?	[51]
Mac-2	1988	47 M	?	–	?	CVCL_H632	?	[52]
*MAC-2B*	1989	47 M	?	–	?	CVCL_H638	DSMZ	[46]
MH-1	1984	61 M	Bone Marrow	?	?	CVCL_H639	?	[53]
SU-DHL-3	1974	35 M	Pleural Effusion	?	?	CVCL_W769	ATCC/DSMZ	[41]
SU-LL-1	1993	?M	?	?	?	CVCL_B5D7	?	[54]
USP-91	1993	14 M	Pleural Effusion	?	?	CVCL_H634	?	[55]

*MAC-2A and MAC-2B are sublines of MAC-2

In this study, we established and characterized an ALCL cell line, PTCL-S1, which has been derived from patient tumour. This cell line has been shown to retain genomic and immunohistochemical profiles of the original tissue, i.e. *TP63*-*TBL1XR1* gene fusion, *NOTCH1* and *AKT1* mutations, CD30 and CD3 positive, CD79a negative. In keeping with previous studies, *NOTCH1* mutations have also been described in ALCL. In addition, *NOTCH1* mutations have been reported in other clinical cases. Larose et al. previously reported *NOTCH1* T349P variant in 12% of their ALCL samples, and this variant was found to confer growth advantage in their study [29]. In another report by Zhong et al., they found that *NOTCH1* was the second most common mutation (22.7%) among ALK-negative patients in their cohort [30]. It has also shown to be responsive to contemporary ALCL treatment agents, including doxorubicin, etoposide and gemcitabine. Taken together, this indicates that PTCL-S1 can be useful as a *TP63*-rearranged ALK-negative ALCL in vitro model to investigate new treatment candidates as well as to better understand the genetic and molecular features of ALK-negative *TP63*-rearranged ALCL.

In conclusion, we established an ALK-negative ALCL PDX and cell line model harbouring *TP63* rearrangement that can not only recapitulate the original characteristics of the patient tumour, but also propagate in vivo. This model shows promise to be used in the understanding of disease mechanism of *TP63*-rearrangement associated ALCL as well as developing new treatments to better patient outcomes.

## Supplementary Information

Below is the link to the electronic supplementary material.

Supplementary file1 (XLSX 10 KB)
